# Efficient and Stable Photoassisted Lithium-Ion Battery Enabled by Photocathode with Synergistically Boosted Carriers Dynamics

**DOI:** 10.1007/s40820-024-01570-7

**Published:** 2024-11-27

**Authors:** Zelin Ma, Shiyao Wang, Zhuangzhuang Ma, Juan Li, Luomeng Zhao, Zhihuan Li, Shiyuan Wang, Yazhou Shuang, Jiulong Wang, Fang Wang, Weiwei Xia, Jie Jian, Yibo He, Junjie Wang, Pengfei Guo, Hongqiang Wang

**Affiliations:** 1 State Key Laboratory of Solidification Processing, Center for Nano Energy Materials, School of Materials Science and Engineering, Northwestern Polytechnical University and Shaanxi Joint Laboratory of Graphene (NPU), Xi’an, 710072 People’s Republic of China; 2 State Key Laboratory of Solidification Processing, School of Materials Science and Engineering Department, Northwestern Polytechnical University, Xi’an, 710072 People’s Republic of China; 3 State Key Laboratory of Solidification Processing, Center of Advanced Lubrication and Seal Materials, School of Materials Science and Engineering, Northwestern Polytechnical University, Xi’an, 710072 People’s Republic of China; 4 Research and Development Institute of Northwestern Polytechnical University in Shenzhen, Shenzhen, 518063 People’s Republic of China; 5 Key Laboratory of Applied Surface and Colloid Chemistry, Shaanxi Key Laboratory for Advanced Energy Devices, Shaanxi Engineering Lab for Advanced Energy Technology, School of Materials Science and Engineering, National Ministry of Education, Shaanxi Normal University, Xi’an, 710119 People’s Republic of China

**Keywords:** Photoassisted lithium-ion batteries, Bulk heterojunction, Carrier dynamics, TiO_2_ nanofiber, Plasmonic metal nanocrystals

## Abstract

Efficient and stable photocathodes with versatility are of significance in photoassisted lithium-ion batteries (PLIBs), while there is always a request on fast carrier transport in electrochemical active photocathodes. Present work proposes a general approach of creating bulk heterojunction to boost the carrier mobility of photocathodes by simply laser assisted embedding of plasmonic nanocrystals. When employed in PLIBs, it was found effective for synchronously enhanced photocharge separation and transport in light charging process. Additionally, experimental photon spectroscopy, finite difference time domain method simulation and theoretical analyses demonstrate that the improved carrier dynamics are driven by the plasmonic-induced hot electron injection from metal to TiO_2_, as well as the enhanced conductivity in TiO_2_ matrix due to the formation of oxygen vacancies after Schottky contact. Benefiting from these merits, several benchmark values in performance of TiO_2_-based photocathode applied in PLIBs are set, including the capacity of 276 mAh g^−1^ at 0.2 A g^−1^ under illumination, photoconversion efficiency of 1.276% at 3 A g^−1^, less capacity and Columbic efficiency loss even through 200 cycles. These results exemplify the potential of the bulk heterojunction strategy in developing highly efficient and stable photoassisted energy storage systems.

**Supplementary Information:**

The online version contains supplementary material available at 10.1007/s40820-024-01570-7.

## Highlights

- Developing a universal bulk heterojunction strategy to create oxygen vacancies by embedding laser-manufactured metal nanocrystals into the TiO_2_ matrix.
- Proposing a new mechanism based on plasmonic-induced hot electron injection and enhanced conductivity from Schottky contact-derived oxygen vacancies.
- Establishing several benchmark values for the performance of TiO_2_-based photocathodes in photoassisted lithium-ion batteries.

**Supplementary Information:**

The online version contains supplementary material available at 10.1007/s40820-024-01570-7.

## Graphical abstract

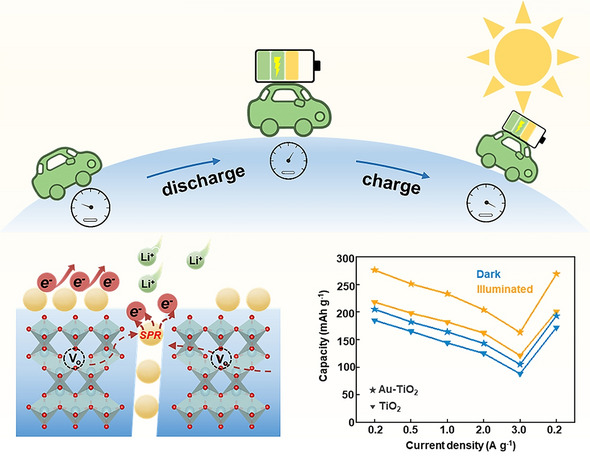

## Introduction

Developing solar energy supplies are essential in addressing the challenges posed by the energy crisis and combating energy poverty in future societies [1–3]. Traditionally, the common approach has been to integrate photovoltaic cells and lithium-ion batteries as off-grid energy storage devices, while these systems encounter difficulties such as ohmic losses, voltage mismatching, and packaging limitations, thereby hindering the further development of this field [4–7]. Photoassisted battery that can combine photoelectronic capabilities with energy storage in a single device, integrates the functions of capturing and utilizing light energy for both generating electrons and storing energy within the battery. Such unique design allows the battery to significantly enhance its discharge capacity when exposed to illumination. In addition, this integration also enables miniaturization, making the battery more compact and suitable for various applications [8–12]. Among the various types of photoassisted batteries, photossisted Li-ion batteries (PLIBs) have attracted considerable attention due to their high energy density [13–17]. In PLIBs, the photoactive cathode serves as a dual function by absorbing sunlight to generate additional electrons (e^−^) and providing a suitable structure for the rapid (de)-insertion of Li^+^ ions, thereby contributing significantly to the high capacity and ideal potential of the battery while playing a crucial role in both photoelectric conversion and energy storage [18–20].

Semiconductor materials that have the potential to meet the aforementioned requirements often suffer from rapid recombination of photogenerated carriers due to their inappropriate band structures [21–23]. To address this issue, a common approach is to construct a hybrid electrode by combining a photosensitizer and a conductive agent for enhanced electron transport. However, such physical mixing could introduce defects, inhomogeneity and multi-interface barriers, leading to electron loss and recombination that ultimately limit the efficiency of photoelectric conversion [24–26]. Creating a heterojunction through elaborately interfacial alignment of two different semiconductor materials offers an effective strategy to enhance charge separation and optimize electron transport path for efficient PLIBs [27, 28]. Furthermore, heterojunctions can be employed to merge materials with different photoelectric and energy storage properties, meeting the requirements in creation of dual-function electrodes [29–31]. A notable example is that the design of the SnO_2_/TiO_2_ heterojunction in which photogenerated electrons could quickly transfer to SnO_2_ due to its more positive conduction band potential compared to TiO_2_ [32]. The presence of holes then promoted the insertion of hat additional Li^+^ ions into TiO_2_ to maintain charge balance, contributing thus to high energy density of the system. It is worth noting that while many studies have focused on such planar heterojunctions, this configuration often results in reduced battery capacity density due to the increased mass of the electrode material. In contrast, the bulk heterojunction configuration offers significant advantages by enabling sufficient photon harvesting and efficient charge extraction without the additional weight. More importantly, bulk heterojunctions in photocathodes not only accelerate electrochemical reaction kinetics by providing additional active sites, but also extend the operating potential window by enabling a broader range of accessible electrochemical reactions. This thus highlights the need to explore bulk heterojunctions. TiO_2_, as a typical low-cost semiconductor material, can produce photogenerated charge carriers when exposed to light, making it suitable as a photoactive cathode. However, the practical application of TiO_2_ is limited by its wide band gap, which results in low photoelectronic conversion efficiency [33, 34]. Given that one-dimensional (1D) nanomaterials possess a large draw ratio and specific electronic transport channels, TiO_2_ nanofibers are conducive to the fast electron transfer and considered as a promising candidate to construct bulk heterojunctions [35, 36]. Unfortunately, it is yet a challenge currently to search for efficient objects embedded into the TiO_2_ nanofibers that could simultaneously boost photoelectron conversion and energy storage for PLIBs [37–39].

Herein, a universal bulk heterojunction strategy is proposed to synergistically improve the carrier dynamics of metal oxide-based photocathodes, exemplified by embedding plasmonic nanocrystals into 1D TiO_2_ nanofibers. A series of plasmonic metals (Au, Ag, and Pt) with well-defined size were generated via pulsed laser irradiation of their bulk counterparts. Subsequently, a modified electrospinning method was employed to enable the plasmonic metals embedded into TiO_2_ matrix to form a metal-TiO_2_ bulk heterojunction. Experimental and theoretical investigations suggest that the localized surface plasmon resonance (LSPR) effect of metal nanocrystals enhanced optical absorption of the bulk heterojunction photocathode in visible light. More importantly, the embedding of the plasmonic metals induced the formation of oxygen vacancies (O_vac_) within TiO_2_ matrix, resulting in the improved carrier dynamics due to the hot electron injection from metal to TiO_2_ as well as the boosted intrinsic conductivity of TiO_2_ after Schottky contact. These merits endowed TiO_2_-based PLIBs with efficient photocharging separation and transportation in light charging process. Exemplified by the representative Au–TiO_2_ photocathode, we achieved an impressive capacity of 276 mAh g^−1^ (at 0.2 A g^−1^) under illumination, accompanied by a substantial photoconversion efficiency of 1.276% at 3 A g^−1^, as well as no capacity and Columbic efficiency loss even through 200 cycles, which sets a new benchmark among TiO_2_-based LIBs reported thus far. Such a universal bulk heterojunction strategy of embedding plasmonic metals into TiO_2_ matrix will pave a new way to advance highly efficient and stable photoassisted energy storage systems.

## Experimental Section

### Preparation of Au Nanocrystal Colloidal Solution

A bulk Au plate was placed in a customized reaction cell with 10 mL ethyl alcohol for laser irradiation. An Nd: YAG non-focusing pulsed laser (repetition rate: 10 Hz, pulse width: 8 ns, beam diameter: 8 mm) with 1064 nm was used to irradiate the Au in ethyl alcohol under continuous ultrasonication in N_2_ environment. The laser fluence varied in 0.8 ~ 1.0 J/pulse cm^−2^. After irradiation, the Burgundy Au–ethanol nano-colloidal solution was obtained.

### Preparation of Au–TiO_2_ and TiO_2_ Nanofiber

In a modified electrospun procedure, 1 g tetrabutyl titanate was mixed with 2 mL Au–ethanol solution, 2 mL acetic acid and 1 mL N, N-dimethylformamide (DMF), and then 0.4 g PVP was dissolved in the mixture and continuously stirred for 8 h. The electrospinning process was set at a feed rate of 0.3 mL h^−1^ with a voltage of 14 kV. The obtained fibers were protoxidized in a vacuum oven at 150 °C for 12 h and calcinated at 500 °C for 4 h with a heating rate of 2 °C min^−1^ to obtain Au–TiO_2_ nanofibers. The compared TiO_2_ nanofibers were obtain in the same process with pure ethanol instead of Au-ethanol solution.

### Materials Characterization

The elemental content of synthesized materials was determined by ICP-OES (Agilent 725). The crystal structures were characterized by an X-ray diffractometer (Rigaku D/Max-3c: Cu K*α*, *λ* = 0.154 nm) and Raman spectra (Renishaw invia, 532 nm). The Rietveld refinement was performed using the GSAS + EXPGUI suite. The morphology was observed by using scanning electron microscopy (TM-3000) and transmission electron microscopy (FEI-Talos F200X). The EDX elemental mapping and 3D reconstruction were also obtained by the Talos F200X TEM with detectors. The elements and chemical states were carried out on X-ray photoelectron spectroscopy and ultraviolet photoelectron spectroscopy (AXISULTRA, Kratos Analytical Ltd.) The defect mode was investigated by ESR (Bruker Magnettech ESR5000). The absorption of fibers was measured by UV–Vis spectrophotometer (Bruker AVANCE III 600). Carrier behavior was analyzed by photoluminescence (PL) and time-resolved photoluminescenc (TRPL) (DeltaFlex) with a Xenon lamp.

### Design and Assembly of Photo-LIBs

The photocathode was composed of 90 wt% Au–TiO_2_ nanofiber and 10 wt% polyvinylidene fluoride (PVDF) in N-methyl l-2-pyrrolidione (NMP) and coated on carbon paper (the loading was 0.4–0.6 mg cm^−2^). The CR2032-type coin cell was designed by making a ~ 8 mm hole on cathode shell and sealed with a transparent PET window using EPOXY for light illumination. The designed coin cells were assembled in an Ar-filled glove box (< 1 ppm of H_2_O and O_2_) with the 1 M LiPF_6_ (EC: DMC) electrolyte, the separator of a celgard 2400, Au–TiO_2_ photocathode and a high-purity lithium sheet as counter electrodes.

### Electrochemical Characterization of Photo-LIBs

A LAND battery tester was employed for investigating the galvanostatic charge/discharge performance, rate capabilities, and cycle performances. A Solartron 1260–1460 E machine was used for testing CV (at a voltage of 1–3 V, scan rate is 0.5 mV s^−1^) and EIS (at a voltage amplitude 10 mV, and the frequency ranges from 10^5^ to 10^−1^ Hz). Conductivity was measured by double electric four-probe tester (RTS-9) at different pressure. All the cell tests were achieved in the dark and illumination conditions by using Xe lamb incubator (BOLING BTC-400). For the illuminated condition, a Xenon lamp light source with wavelengths ranging from 400 to 1100 nm and a light intensity of 1 sun (100 mW cm^−2^) was employed.

### Fabrication of PDs and Electrical Measurements

The photoelectric properties are measured by a three-electrode system. The photoelectrode was constructed by an electrospun method with FTO substrate, the reference electrode was Ag/AgCl and the platinum wire served as a counter electrode. I-V and I-t curves were measured by using Solartron 1260–1460 E machine.

### Li^+^ Diffusion Coefficient Calculation

Li^+^ diffusion coefficient was calculated using EIS curves:

1 $$D = \frac{{{\text{R}}^{{2}} {\text{T}}^{{2}} }}{{{\text{2A}}^{{2}} {\text{n}}^{{4}} {\text{F}}^{{4}} {\text{C}}^{{2}} \sigma^{{2}} }}$$

where *R* is the gas constant, *T* is the room temperature in Kelvin, *A* is the surface area of the electrode, *n* is the number of electrons per molecule involved in the charge and discharge process, F is the Faraday constant, *C* is the concentration of Na + in the NMA and NMA@AlO_*x*_ electrodes, and *σ* is the slope of the line Z′ ∼ *ω*^−1/2^.

### Photoconversion Efficiency (%)

Photoconversion efficiency was calculated as the above process at different current densities according to the Folume (2):

2 $${\text{Photo}} - {\text{conversion}}\;{\text{efficiency }}\left( \% \right) = \frac{{E_{2} - E_{1} }}{{100 \left( {{\text{mW}}\;{\text{cm}}^{ - 2} } \right) \times S \times h}} \times 100\%$$

where *E*_1_ and *E*_2_ is the charging energy under dark and illumination, respectively, *S* is the area of the optical window (0.50 cm^2^), and *h* is the illuminated time of the photo-LlBs.

### Simulation Method

Plasmonic near-field maps were simulated with commercial Ansys Lumerical software (FDTD). The diameter of Au nanocrystals was fixed at 10 nm. The scale of TiO_2_ was set to be 60 nm × 60 nm × 15 nm. The refractive index of Au–TiO_2_ was assumed to be 1.0, and the dielectric constant of Au was from the Johnson and Christy database. The excitation wavelength of a plane wave light source is 350–800 nm.

#### Density Functional Theoretical Calculation

All theoretical calculations were performed using the Vienna ab initio simulation package (VASP) in this work [40]. The projector-augmented wave (PAW) pseudopotentials were used to describe the interactions between valence electrons and ionic cores [41]. The Perdew-Burke-Ernzerhof (PBE) functional of the generalized gradient approximation (GGA) was employed to account for electronic exchange and correlation [42]. A plane wave basis set with a cutoff of 400 eV was adapted to expand the wave functions. To simulate the TiO_2_ and Au cluster interface, the (101) surface with O_4_ termination of Ti_24_O_48_ slab with 12 layers and Au_4_ cluster were used. The bottom six layers of the Ti_24_O_48_ slab were fixed in their bulk positions. The remaining TiO_2_ layers and Au_4_ clusters were relaxed using the conjugate gradient algorithm until the maximum force on a single atom was less than 0.05 eV Å^−1^. The Г-centered k-point sampling of 2 × 3 × 1 was done by the Monkhorst–Pack method [43]. For the Ti_24_O_48_/Au_4_ slab, the vacuum region of 15 Å was set to avoid the interactions between neighboring layers. The formation energy of oxygen vacancy in the Ti_24_O_48_ slab and the Ti_24_O_48_/Au_4_ interface were calculated according to the equations:

3 $${\text{E}}_{{\text{o - vacanvcy}}} {\text{ = E(Ti}}_{{{24}}} {\text{O}}_{{{47}}} {)}{ - }{\text{E(Ti}}_{{{24}}} {\text{O}}_{{{48}}} {\text{) + E(O)}}$$

4 $${\text{E}}_{{\text{o - vacanvcy}}} {\text{ = E(Ti}}_{{{24}}} {\text{O}}_{{{47}}} {\text{/Au}}_{{4}} {)}{ - }{\text{E(Ti}}_{{{24}}} {\text{O}}_{{{48}}} {\text{/Au}}_{{4}} {\text{) + E(O)}}$$

where *E*(Ti_24_O_47_) and *E*(Ti_24_O_47_/Au_4_) are the total energy of Ti_24_O_48_ slab and Ti_24_O_48_/Au_4_ interface with one oxygen vacancy, *E*(Ti_24_O_48_) and *E*(Ti_24_O_48_/Au_4_) are the total energy of perfect slab and interface. *E*(O) is taken from the half of total energy of free oxygen molecule, which is corresponding to O-rich condition. The charge density difference between the TiO_2_ slab and Au_4_ cluster were obtained using the VASPKIT code [44]. The yellow region represents charge accumulation and the blue region charge depletion. The crystal structure of model was drawn using the VESTA code [45].

## Results and Discussion

### Photocharging Mechanism of Bulk Heterojunction Photocathodes

**Fig. 1:**
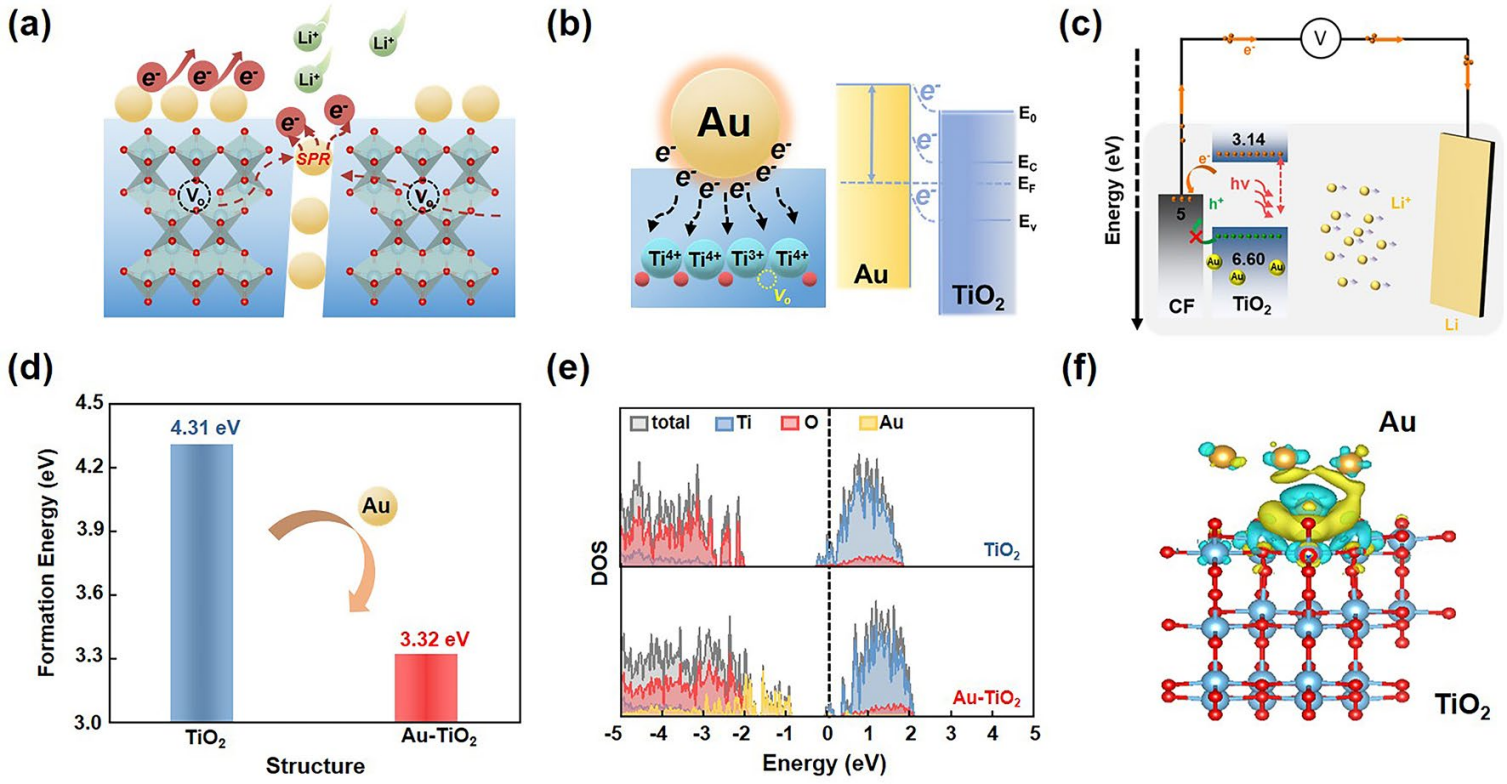
TiO_2_-based photocathodes applied in PLIBs by constructing bulk heterojunctions with laser-embedded metal nanocrystals. **a** Schematic illustration of the synergistically improving of carriers dynamics in TiO_2_ photocathode enabled by embedding plasmonic nanocrystals. **b** Schematic diagram of band structure and electron transfer at the interface of metal and TiO_2_. **c** Schematic illustration of the proposed photocharging/discharging mechanism of metal-TiO_2_ PLIBs. **d** DFT-calculated formation energy of oxygen vacancy for TiO_2_ and Au–TiO_2_, respectively. **e** DFT-calculated DOS for TiO_2_ and Au–TiO_2_, respectively. **f** Charge density difference of Au–TiO_2_

The proposed TiO_2_-based bulk heterojunction was achieved through the embedding of plasmonic metals, illustrating in Fig. 1a. It can be seen that the laser-generated metal nanocrystals are distributed within the TiO_2_ matrix, forming a series of local Schottky contacts between the metal and TiO_2_ (Fig. 1b). Accompanied by the formation of O_vac_, the intrinsic conductivity and the light absorption ability of the TiO_2_ matrix will significantly increase due to the barrier reduction induced hot electrons injection from metal to TiO_2_. The photodischarging/charging process of the subsequently assembled PLIBs is schematically depicted in Fig. 1c. Under illumination, photogenerated electrons are excited and transferred to the conduction band (CB) of the bulk heterojunction photocathode, and then transported to carbon paper (CP) with a small barrier. Meanwhile, the photogenerated holes from valence band (VB) are hindered by CP due to a large interfacial extraction barrier. This combined behavior of electron transfer and hole blocking enables the photoassisted charging process in the PLIBs.

In order to elucidate the microstructure evolution in TiO_2_-based bulk heterojunctions, the density functional theoretical (DFT) calculations were performed on the optimized models of TiO_2_ and its bulk heterojunction (Fig. S1). Exemplified by the representative Au-TiO_2_ bulk heterojunction, the formation energy of O_vac_ in Au–TiO_2_ (3.32 eV) is lower than that of TiO_2_ (4.31 eV), indicating that embedding Au nanocrystals into TiO_2_ induces the generation of O_vac_ (Fig. 1d). This is attributed that the outermost electrons of Au 4*f* are slightly transferred to Ti 2*p* upon contact between Au and TiO_2_, leading to the formation of O_vac_ in TiO_2_ matrix to balance charge, thereby improved electron and ion conductivity. Density of states (DOS) calculations were performed to further investigate the effects of Au on electronic structure of TiO_2_. As shown in Fig. 1e, the defective energy levels at around ~ 2 eV corresponding to Au-vacancy interactions are able to facilitate fast conductivity. The position of the valence edge in Au–TiO_2_ is shifted above the Fermi level compared to TiO_2_, indicating a decreased band gap in Au–TiO_2_. This reduction in the band gap could enhance photoabsorption and excitation of electrons under illumination. Furthermore, the charge density difference (CDD, Fig. 1f) reveals that the electron density at the Au–TiO_2_ interface is significantly higher than that in TiO_2_ matrix, the Schottky contact and defect state can reduce the electronic affinity and be conducive to electron transport, suggesting that the embedding of Au nanocrystals in TiO_2_ promotes electron transfer and Li^+^ transport and insertion [46–48]. These DFT results demonstrate that constructing bulk heterojunctions by embedding metal nanocrystals in TiO_2_ matrix is conducive to enhance the photoelectric properties of PLIBs.

### Construction and Characterization of Au–TiO_2−x_ Bulk Heterojunctions

Experimentally, the Au–TiO_2_ bulk heterojunction was constructed by a modified electrospinning method (Fig. S2). Briefly, the Au nanocrystals were first manufactured in ethanol through the pulsed laser irradiation of a gold target. This process resulted in the formation of a brownish-red colloid solution with clear Tyndall scattering, yielding well-dispersed Au nanocrystals with an average diameter of 9.51 nm (Fig. S3). The crystal structure of the as-prepared Au nanocrystals was determined by high-resolution transmission electron microscopy (HRTEM), exhibiting a lattice spacing of 2.35 Å which corresponds to the typical plane (111) of Au (Fig. S3b). The as-prepared Au colloidal solution was subsequently employed to blend with the TiO_2_ precursor and then subjected to electrospinning onto a silicone paper substrate under high voltage. Such spinning enabled the in situ embedding of laser-generated Au nanocrystals within the TiO_2_ nanofibers, resulting in the formation of the Au–TiO_2_ bulk heterojunction.

**Fig. 2:**
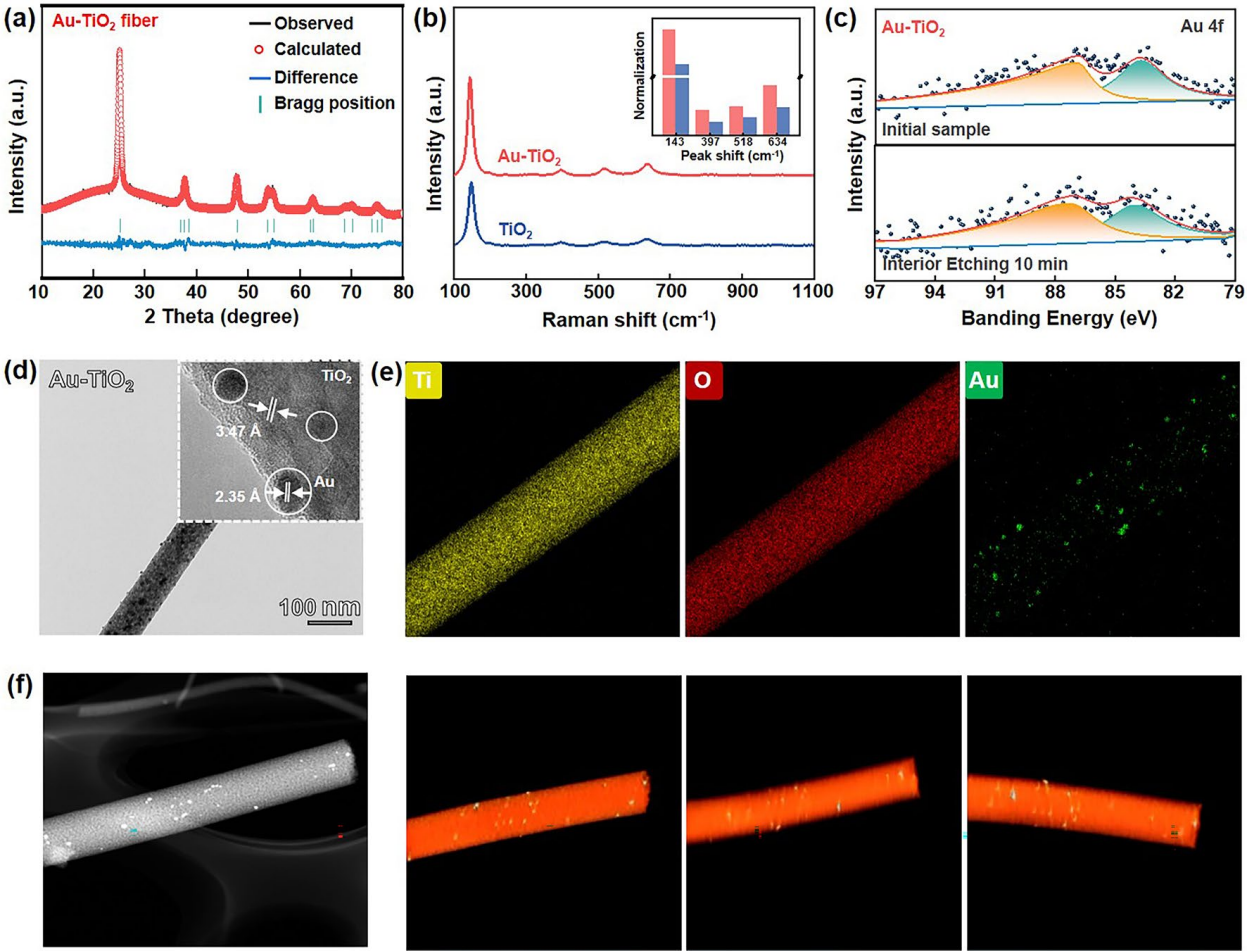
Structure and morphology characterization of the prepared TiO_2_ and Au–TiO_2_ nanofibers. **a** XRD pattern and Rietveld plots of Au–TiO_2_. **b** Raman spectra of TiO_2_ and Au–TiO_2_ (insert, normalization peak). **c** Au 4*f* XPS spectra of the prepared Au–TiO_2_ with different etching times of 0 and 10 min. **d** TEM and HRTEM images of Au–TiO_2_. **e** EDX mappings (Ti, O and Au elements) of Au–TiO_2_ and **f** HADDF and 3D TEM reconstruction images of Au–TiO_2_

**Fig. 3:**
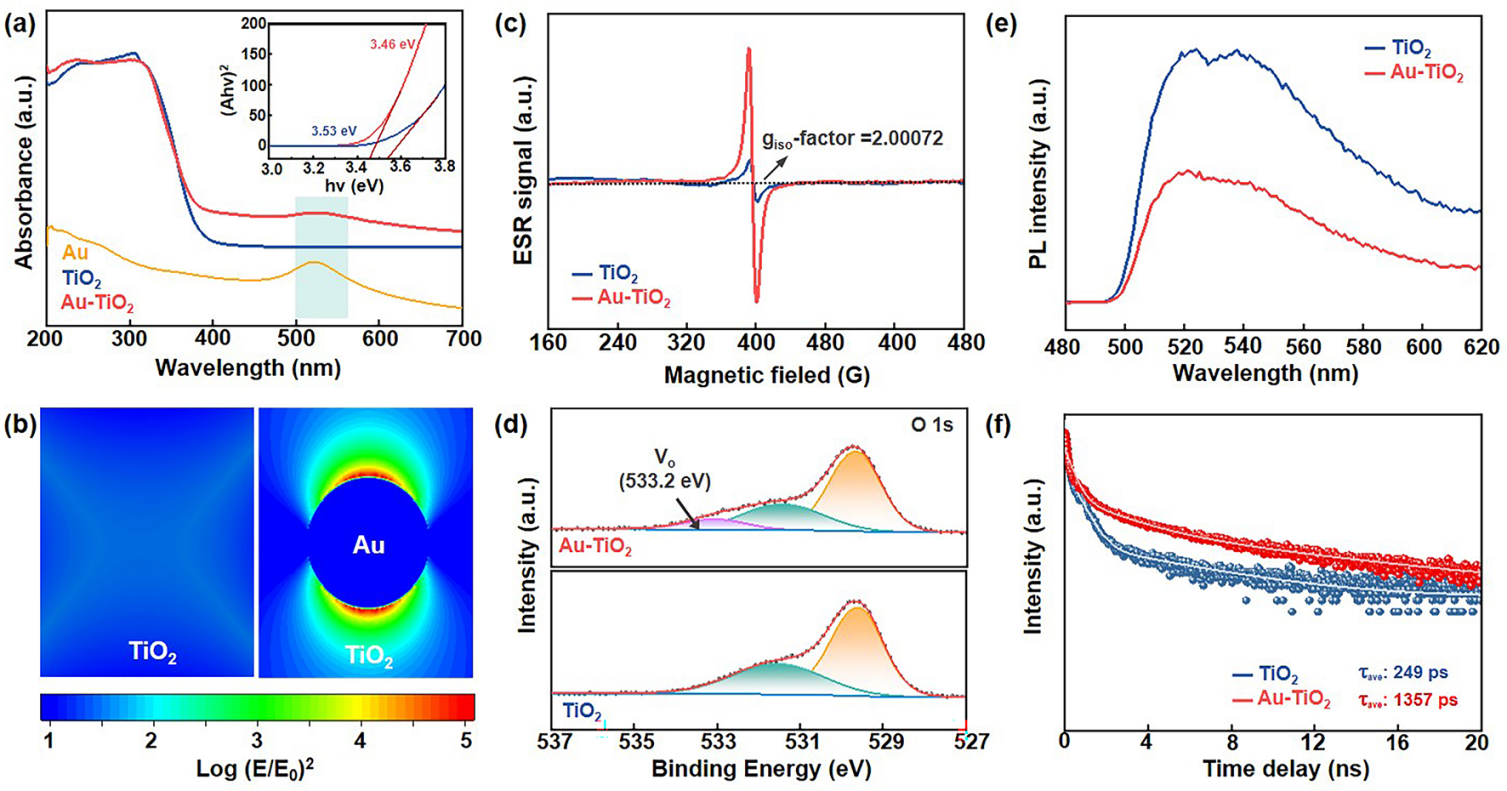
Photoelectric properties and enhancement mechanisms of Au–TiO_2_. **a** UV–vis absorption spectra of the Au nanocrystals, TiO_2_ and Au–TiO_2_ (insert, the corresponding Tauc plot of the three samples). **b** FDTD simulations of TiO_2_ and Au–TiO_2_, E_0_ and E represent the incident and localized electric field, respectively. **c** ESR spectra of TiO_2_ and Au–TiO_2_. **d** XPS spectra of O 1*s* peaks of TiO_2_ and Au–TiO_2_. **e** PL spectra and **f** TRPL spectra of TiO_2_ and Au–TiO_2_

To determine the crystal structure of obtained Au-TiO_2_ nanofiber, the X-ray powder diffraction (XRD) and the XRD Rietveld refinement were conducted using the GSAS + EXPGUI method, and corresponding XRD patterns and refinement results are shown in Figs. 2a, S4 and Table S1. The XRD pattern of Au–TiO_2_ corresponds to that of the pristine TiO_2_ nanofiber, indicating that both belong to anatase phase in the *I41/amd* space group (*a* = *b* = 3.7779 Å, *c* = 9.4559 Å, *R*_wp_ = 7.23%, *R*_p_ = 5.52% and χ^2^ = 1.6680). The similar lattice parameters suggest that the crystal structure of TiO_2_ remains unchanged when Au nanocrystals are embedded into TiO_2_ fibers. However, no diffraction peaks for Au are observed in Au–TiO_2_, likely due to the low Au content of only 4.1%, as determined by inductively coupled plasma analysis (ICP, Table S2). It is worth noting that Au nanocrystals enable a prominent surface Raman enhancement effect for oxides. Therefore, Raman spectroscopy was employed to investigate the presence of Au nanocrystals. As shown in Fig. 2b, the Au–TiO_2_ spectrum shows significantly enhanced peaks corresponding to the Ti–O bond vibrations in anatase TiO_2_, located at 143, 400, 517, and 638 cm^−1^, respectively. The normalized bar graph in the inset of Fig. 2b provides a clear visual comparison between the two samples, clearly demonstrating the existence of Au nanocrystals in Au–TiO_2_. The X-ray photoelectron spectroscopy (XPS, Figs. 2c and S5) characterization was carried out to further analyze the element type and valence state. To investigate the presence of Au nanocrystals within Au–TiO_2_ nanofibers, the Au 4*f* orbital was analyzed using the Ar^+^ surface etching method. Interestingly, the signal of the Au (0) at 84.2 and 87.8 eV can be detected in the Au–TiO_2_ nanofibers even after etching 10 min, demonstrating Au nanocrystals enter into the TiO_2_ matrix. In the Ti 2*p* orbital (Fig. S5), both the Au-TiO_2_ and TiO_2_ nanofibers exhibit two sharp peaks at 458.4 and 464.2 eV, which are consistent with the Ti^4+^. Nevertheless, in comparison with the pure TiO_2_, one additional peak at 462.0 eV corresponding to Ti^3+^ appears in Au–TiO_2_. This is attributed to surface electron transfer resulting from the embedding of Au nanocrystals and might balance the formed O_vac_. Notably, the XPS spectra of the O 1*s* in Au–TiO_2_ show a distinct O_vac_ peak at 533.4 eV (Fig. 3d). These results suggest that Au nanocrystals successfully enter the TiO_2_ nanofiber matrix during the electrospinning process, along with the formation of O_vac_.

A series of morphology characterizations were further carried out to visualize the distribution of Au nanocrystals in TiO_2_ nanofiber. The scanning electron microscopy (SEM) images show that both samples present fibrous shapes with a diameter of ~ 100 nm (Fig. S6), and the Au nanocrystals did not affect the microstructure of TiO_2_ nanofibers. It should be noted that the Au–TiO_2_ sample prepared by traditional wet chemical methods is subjected to a staccato state owing to the usually grafted ligands on Au nanocrystals surface (Fig. S7). As shown in Figs. 2d and S8, it is clearly seen that the lattice fringes of Au nanocrystals with an inner-plane spacing at 2.35 Å (111) are surrounded by TiO_2_ grains with the typical inner-plane spacing of 3.47 Å in (101), elucidating the location and distribution in TiO_2_ nanofibers. Furthermore, the Au nanocrystals were found to be dispersed into the TiO_2_ nanofiber, as evidenced by the EDX mappings of Ti, O, and Au elements shown in Figs. 2e and S9. In addition, 3D TEM reconstruction shows in Fig. 2f and was employed to further confirm that Au nanocrystals indeed enter the polycrystalline TiO_2_ nanofiber by a multi-angle observation. All the above structural and morphological results clearly indicate that the Au–TiO_2_ nanofiber with local bulk heterojunctions has been successfully constructed without any damage to the TiO_2_ matrix.

### Photoelectric Properties of Au–TiO_2_ Photocathodes

The photosensitivity and photocarrier separation/transport for the TiO_2_ and Au–TiO_2_ photoelectrodes were studied in a photodetector system, as depicted in Fig. S10. As expected, the Au–TiO_2_ photoanodes exhibited a stronger current response compared to TiO_2_ when exposed to 520 nm illumination owing to the increased photocarrier induced by Au nanocrystals, evidenced by the current–voltage (*I*–*V*) curves under illumination (Fig. S11). Furthermore, the current–time (*I*–*t*) measurements were conducted at a bias voltage of 1 V under periodic light and dark conditions, demonstrating that Au–TiO_2_ photoanodes are capable of generating a large photocurrent (Fig. S12). The improved photoelectric performance can be attributed to the unique microstructure formed between Au and TiO_2_, leading to the plasmonic-induced hot electron injection from metal to TiO_2_, as well as the formation of oxygen vacancies after Schottky contact [49, 50]. The SPR effect of Au nanocrystals is evident in the ultraviolet–visible (UV–vis) spectra of Au–TiO_2_ shown in Fig. 3a, where a distinct absorption peak is observed at ~ 520 nm. Also, the embedding of Au nanocrystals and the formation of oxygen vacancies in TiO_2_ matrix can decrease CB and reduce the band gap of TiO_2_ nanofibers from 3.53 to 3.46 eV, demonstrating the enhanced light absorption ability in visible light range for the TiO_2_ photoelectrodes [51–53]. In Fig. 3b, the simulated electric field intensity around TiO_2_ and Au–TiO_2_ of the finite difference time domain (FDTD) method reveals that the electric field intensity of Au–TiO_2_ is enhanced by approximately 6 times in the vicinity of the Au nanocrystals, creating spatially confined “hot spots”. Such enhancement indicates the presence of plasmon-excited hot electrons at the interface of TiO_2_ and Au nanocrystals, contributing to an increase in the amount of charge in the battery system and improving the performance of TiO_2_-based PLIBs [54, 55]. More importantly, according to the ultraviolet photoelectron spectroscopy (UPS) analysis shown in Fig. S13, the valence band (VB) and the conductive band (CB) of Au–TiO_2_ nanofibers are calculated to be 6.60 and 3.14 eV, respectively, indicating that the O_vac_ changes the band structure of TiO_2_ (6.36 and 2.83 eV), which improve the photoassisted battery performance. The energy level arrangement is appropriate and favorable for the transfer of photogenerated electrons into the counter electrode while preventing the propagation of photogenerated holes outward.

The formation of oxygen vacancies was determined by the electron spin-resonance spectroscopy (ESR) shown in Fig. 3c. A stronger signal in Au–TiO_2_ at a g_iso_-factor of 2.00072 indicates the presence of a higher amount of oxygen vacancies (O_vac_) in TiO_2_ due to the Schottky contact induced charge transfer between TiO_2_ matrix and Au nanocrystals. The O_vac_ in Au–TiO_2_ was further confirmed by XPS spectra shown in Fig. 3d. In Au–TiO_2_, an additional broad peak of O 1*s* at 533.2 eV is observed alongside the typical two sharp peaks of TiO_2_ at 531.4 and 529.8 eV, indicating the presence of O_vac_ with a content of approximately 13%. The formation of O_vac_ is attributed to the equilibrium valence state of Ti caused by the embedding of Au nanocrystals and subsequent Schottky contact [56–58]. These O_vac_ in Au–TiO_2_ can provide extra electron states near the E_f_ and enhance the Li^+^ transport and the corresponding charge transfer process, which effectively enhances the electrical conductivity [59–64] and accelerates charge transport of photogenerated electrons, which plays a crucial role in improving the overall photoelectric performance and Li^+^-(de) insertion processes of PLIBs [65, 66]. The steady-state and time-resolved photoluminescence (PL) spectra of TiO_2_ nanofibers with or without Au nanocrystals were further conducted to investigate the charge transport. Figure 3e illustrates a significant quenching of the PL intensity in Au–TiO_2_ compared to pure TiO_2_, indicating the excellent electron extraction ability of Au–TiO_2_ photocathodes. Additionally, the average carrier decay lifetime increased from 249 to 1357 ps in Au–TiO_2_ (Fig. 3f). These results demonstrate that Au nanocrystals facilitate the separation of photogenerated electrons and holes, thereby enhancing the photoelectric properties of TiO_2_ nanofibers. This enhancement is primarily attributed to the synergistic effect of SPR and O_vac_ induced by the constructed Au–TiO_2_ bulk heterojunctions.

### Photoassisted Li-Storage Performance of Au–TiO_2_ Photocathodes

**Fig. 4:**
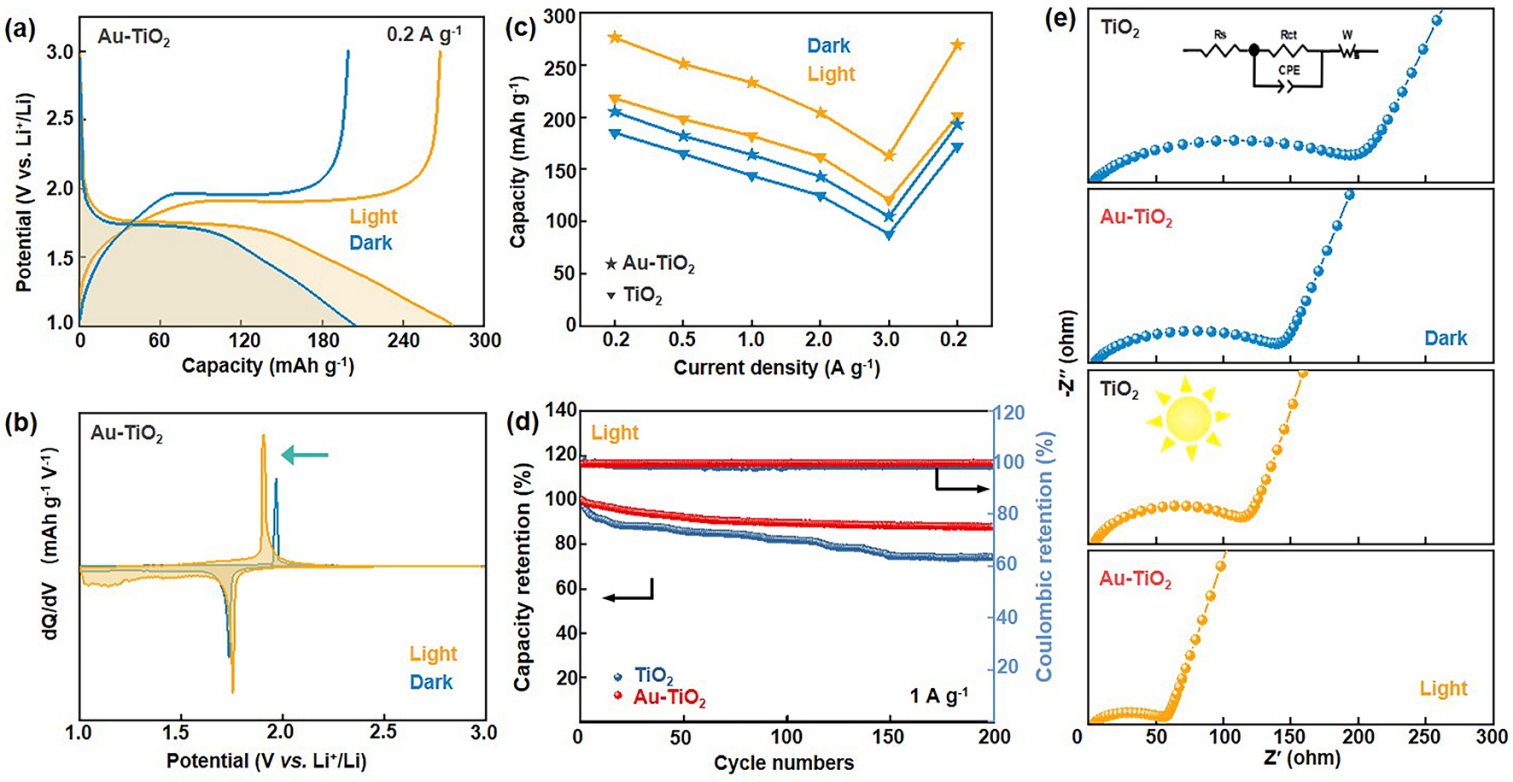
Electrochemical performance of TiO_2_ and Au–TiO_2_ photoassisted batteries. **a** Discharge–charge curves and **b** dQ/dV versus voltage of Au–TiO_2_ sample at 0.2 A g^−1^ in the dark and light condition. **c** Rate performance of TiO_2_ and Au–TiO_2_ at different current densities. **d** Cycling performance and Columbic efficiency of Au–TiO_2_ photocathode at 1 A g^−1^. **e** EIS curves of TiO_2_ and Au-TiO_2_ photoassisted batteries (insert, equivalent circuit diagram)

To assess the photoassisted storage-Li performance of Au–TiO_2_ photocathodes, a CR2032-type coin cell was designed with a ~ 8 mm optical window (sealed with PET film and EXPOY, Fig. S14), and a series of electrochemical measurements were conducted under both light and dark conditions. The results indicate that Au-TiO_2_ photocathode exhibits a distinguishably superior electrochemical performance than TiO_2_ photocathode. The galvanostatic discharging and charging curves of TiO_2_ and Au–TiO_2_ photocathodes were measured at the current density of 0.2 A g^−1^ (Figs. 4a, S15 and Table S3). The capacity of Au–TiO_2_ increased significantly from 203 to 276 mAh g^−1^ when measurements were conducted under dark to light, representing a 35.4% increase. In contrast, the TiO_2_ photocathode exhibits a modest increase from 185 to 218 mAh g^−1^, corresponding to a 17.8% increase. The intuitive discharge and charge voltage variation can be observed in the corresponding dQ/dV curves (Fig. 4b). In the case of Au–TiO_2_, the discharge voltages show enhancement from 1.738 to 1.765 V, while the charge voltages decrease from 1.958 to 1.896 V, indicating the efficient transfer of photogenerative electrons, this behavior is superior to the nominal voltage change observed in the pure TiO_2_ photocathode, as shown in Fig. S16 and detailed in Table S4. Furthermore, the rate and cycling performance of two photocathodes were systematically investigated at different current densities under both light and dark conditions. Figure 4c and Table S3 show that the Au–TiO_2_ cathode exhibits superior rate capacity compared to the TiO_2_ cathode in the dark condition. Specifically, the Au–TiO_2_ cathode maintains discharging capacities of 203, 182, 164, 143, and 105 mAh g^−1^ at different current densities of 0.2, 0.5, 1.0, 2.0, and 3.0 mA g^−1^, respectively. In contrast, the TiO_2_ cathode shows capacities of 185, 165, 144, 125, and 88 mAh g^−1^ at the same current densities. The performance enhancement is primarily attributed to the improved conductivity resulting from the construction of bulk heterojunctions in the Au–TiO_2_ photocathodes. In addition, the discharging capacity of Au–TiO_2_ cathode can recover to 193 mAh g^−1^ when the current density is reduced to 0.2 A g^−1^ again, demonstrating its excellent reversibility. Interestingly, the rate performance of Au–TiO_2_ photocathode can be enhanced under further illustration. The discharging capacities at current densities of 0.2, 0.5, 1.0, 2.0, and 3.0 A g^−1^ for the Au–TiO_2_ cathode increase by 34%, 38%, 42%, 45%, and 55%, respectively. In comparison, the corresponding discharging capacities for the TiO_2_ photocathode only increase by 18%, 21%, 27%, 30%, and 37% under the same conditions. Additionally, the photoconversion efficiency of the Au–TiO_2_ cathode rises from 0.042% to 1.276%, while the TiO_2_ photocathode shows an increase from 0.027% to 0.85% (Fig. S17, Tables S5 and S6).

The test results further demonstrate that the introduction of illumination can improve the electrochemical performance of Au–TiO_2_ PLIBs, which can be attributed to the synergistic enhancement of photocharging separation and transport in photocharging process, accelerated by the plasmonic Au-induced O_vac_. Moreover, the cycling performance measurements depicted in Fig. 4d demonstrate that the Au–TiO_2_ photocathode exhibits excellent capacity retention. The Au–TiO_2_ photocathode still retains 88.0% of its initial capacity (233 mAh g^−1^) after 200 cycles at 1 A g^−1^ in the light condition. Nevertheless, the pure TiO_2_ photocathode shows a lower capacity of 182 mAh g^−1^ with a capacity retention of ~ 75% under the light condition. These results indicate excellent reversibility and long cycle stability for the Au-TiO_2_ photocathode. The kinetic diffusion process of Li^+^ was studied by using electrochemical impedance spectroscopy (EIS) measurements under dark and illumination conditions (Fig. 4e and Table S7). In the dark condition, the Au–TiO_2_ photocathode delivers the lower charge transfer resistance (R_ct_) and the higher Li^+^ diffusion coefficient (D_Li+_, Fig. S18) of 5.84 × 10–8 cm^2^ s^−1^ compared to the TiO_2_ photocathode (Table S7). Meanwhile, it can be seen that the *R*_ct_ of both Au–TiO_2_ and TiO_2_ significantly decreases under illumination, indicating that photo excites charges and accelerates charge transport, particularly in the case of Au–TiO_2_, where it can enhance charge transfer in the TiO_2_ bulk phase. The improvements in capacity, rate performance, and Li^+^/e^−^ transfer behavior evidentially demonstrate that the strategy of constructing bulk heterojunction in TiO_2_ helps enhance the intrinsic photoelectric properties, charge transport behavior, and overall performance of PLIBs.

### Universality of Bulk Heterojunctions and Practical Application

**Fig. 5:**
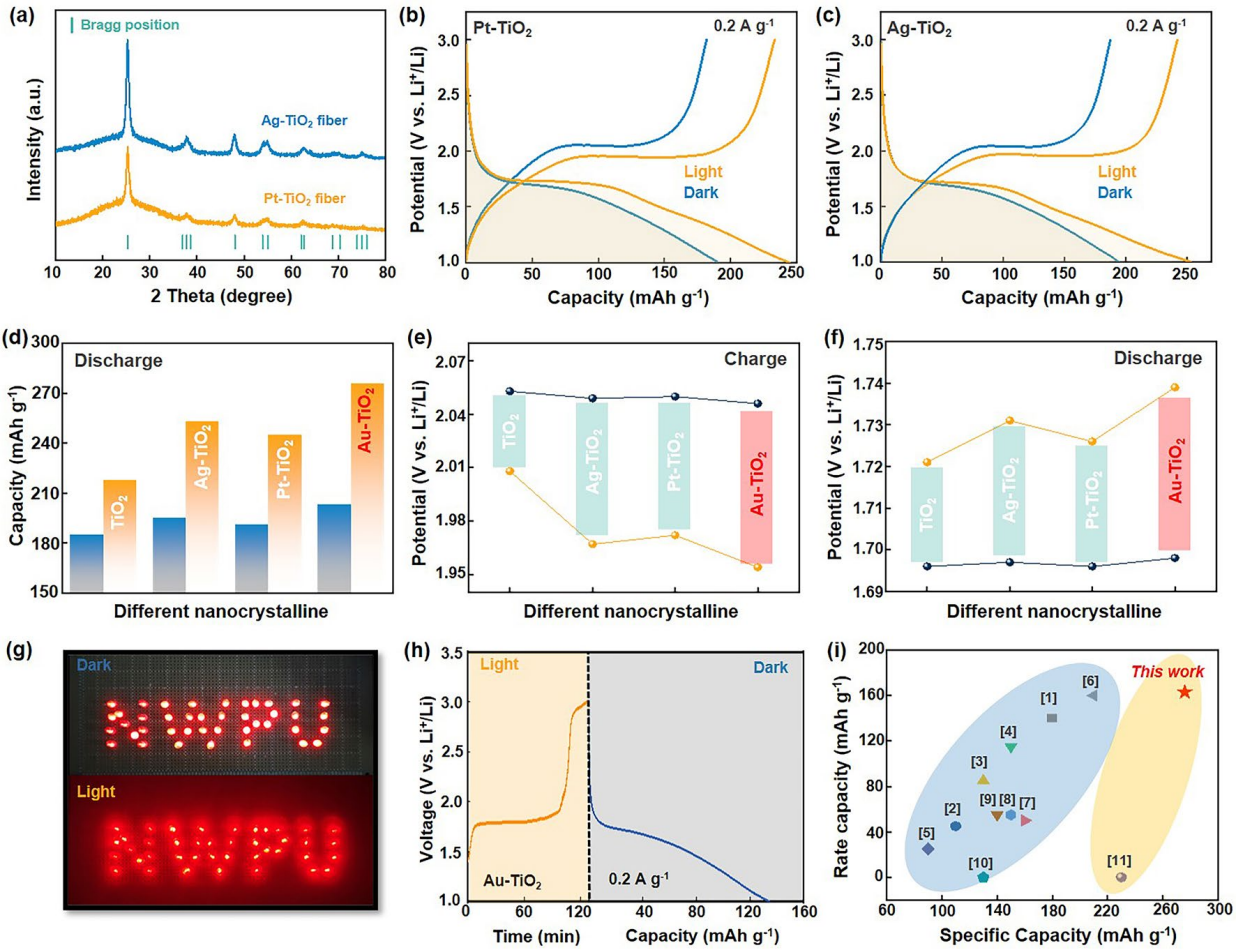
Photocathode expansion and practical application of photoassisted battery. **a** XRD patterns and Rietveld plots of Ag–TiO_2_ and Pt–TiO_2_. **b, c** Discharge–charge curves of Pt–TiO_2_ and Ag–TiO_2_ samples at 0.2 A g^−1^ in the dark and light condition. **d**–**f** Discharge capacities, charge voltage and discharge voltage under illumination for Au–TiO_2_, Ag–TiO_2_ and Pt–TiO_2_. **g** LED arrays in the light and dark with Au–TiO_2_ photocathode. **h** Charge and discharge curves of Au–TiO_2_ without current under illumination. **i** Comparison of specific capacity and rate capacity of different TiO_2_–based materials, the related references are provided in Table S8

The above results suggest the superiority of the bulk heterojunction strategy in improving the optical and electronic properties of the TiO_2_ photocathodes, thereby significantly enhancing photoelectron conversion and energy storage performance of PLIBs. Taking advantage of the universal feature of pulsed laser irradiation for generating metal nanocrystals in liquid, a series of ligand-free metal plasmonic, specifically Pt and Ag nanocrystals with average sizes of 9.50 and 9.20 nm (Fig. S19), respectively, were further prepared in an ethanol-based TiO_2_ precursor solvent. These metal nanocrystals were subsequently embedded in the TiO_2_ fibers to construct bulk heterojunctions, as evidenced by the results in the XRD patterns and UV–vis spectra (Figs. 5a and S20). TEM images further reveal that the Pt and Ag nanocrystals were embedded into the bulk TiO_2_ nanofiber, with sizes remaining about 10 nm, where the Ti and O elements were evenly distributed in the Pt–TiO_2_ and Ag–TiO_2_ nanofibers while Pt and Ag were enriched inside the fibers (Fig. S21). Similarly, such embedding did not change the fiber morphology and diameter (Fig. S22). To investigate the universality of bulk heterojunction strategy, the electrochemical performances of the Pt–TiO_2_ and Ag–TiO_2_ photocathodes were evaluated, as shown in Fig. 5b, c. Compared to pure TiO_2_ at 0.2 A g^−1^ under illumination, the Pt–TiO_2_ and Ag–TiO_2_ exhibit higher specific capacities of 245 and 253 mAh g^−1^, respectively. This suggests that the construction of bulk heterojunctions through the embedding of Pt and Ag nanocrystals has a similar effect on TiO_2_ nanofibers. However, it should be noted that among these bulk heterojunction constructed TiO_2_ photocathodes, the Au–TiO_2_ photocathode exhibits the largest specific capacity, highest discharge voltage, lowest charge voltage, and the largest photoconversion efficiency of 1.276% (Fig. 5d–f).

Given these striking merits, the Au–TiO_2_ photocathodes were further assembled as a PLIB, which can be charged solely by solar energy without any external current, whereas Li^+^ are activated solely by photoelectrons. As illustrated in Fig. 5g, the brightness of the LED arrays increases significantly under illumination, deriving by a series of photoassisted batteries with the Au–TiO_2_ photocathode. Figure 5h shows the photocharging and discharging curve of Au–TiO_2_, featuring a charge voltage plateau at 1.79 V, which reaches the upper voltage limit of 3.0 V after 2 h of illumination. This results in a discharge capacity of 128 mAh g^−1^ at 0.2 A g^−1^ in the dark with the photoconversion efficiency of 0.144%, demonstrating the potential for practical application. In comparison with the reported results (Fig. 5i and Table S8), which is ranked among the top capacity and rate performance of TiO_2_-based electrodes in this work, indicating that these synergistically boosted carriers dynamics tactics can be used as the high-efficiency photocathodes in the photoassisted LIBs.

## Conclusions

In summary, a universal bulk heterojunction strategy was developed to regulate the electronic structure and light harvesting of TiO_2_-based photocathodes for simultaneously enhancing the photocharge separation and transport in light charging process of PLIBs. A series of bulk heterojunctions were obtained by the embedding of laser-manufactured metal nanocrystals into the TiO_2_ nanofibers. The plasmonic metals were determined to be embedded into the bulk TiO_2_, significantly improving the carrier dynamics of the photocathodes through plasmonic-induced hot electron injection from the metal to TiO_2_ and increased conductivity due to Schottky contact-derived oxygen vacancies. As a result, the representative Au–TiO_2_ photocathodes applied in PLIBs proposed several benchmark performances, including the capacity of 276 mAh g^−1^ under illumination, photoconversion efficiency of 1.276% at 3 A g^−1^, as well as less loss in capacity and columbic efficiency over 200 cycles. These findings highlight the potential of the bulk heterojunction strategy for developing highly efficient and stable photoassisted energy storage systems.

## Supplementary Information

Below is the link to the electronic supplementary material.

Supporting Information is available from the file. Including structure models for DFT, TEM and HRTEM images, XRD patterns, XPS spectra, SEM images, EDX mapping images, LSV curves, I-t curves, UPS spectra, optical photograph of photoassisted coin cell, discharge-charge curves, dQ/dV curves, photoconversion efficiency, Z′ vs ω−1/2 curves, UV spectra, refinement results, ICP data, table of performance for battery, EIS fitting data, comparison with other related work and so on. (DOCX 4009 KB)
