# Supplementary material for: Efficient and Stable Photoassisted Lithium-Ion Battery Enabled by Photocathode with Synergistically Boosted Carriers Dynamics
Source: Nanomicro Lett. 2024 Nov 27;17:74. doi: 10.1007/s40820-024-01570-7 (PMC11602903; doi:10.1007/s40820-024-01570-7)
Supplement: Supplementary file 1 — Supporting Information is available from the file. Including structure models for DFT, TEM and HRTEM images, XRD patterns, XPS spectra, SEM images, EDX mapping images, LSV curves, I-t curves, UPS spectra, optical photograph of photoassisted coin cell, discharge-charge curves, dQ/dV curves, photoconversion efficiency, Z′ vs ω−1/2 curves, UV spectra, refinement results, ICP data, table of performance for battery, EIS fitting data, comparison with other related work and so on. (DOCX 4009 KB) [file 40820_2024_1570_MOESM1_ESM.docx]

Supporting Information for

**Efficient and Stable Photo-Assisted Lithium-Ion Battery Enabled by Photocathode with Synergistically Boosted Carriers Dynamics**

Zelin Ma^1,‡^, Shiyao Wang^2,‡^, Zhuangzhuang Ma^5^, Juan Li^3^, Luomeng Zhao^1^, Zhihuan Li^3^, Shiyuan Wang^1^, Yazhou Shuang^1^, Jiulong Wang^1^, Fang Wang^1^, Weiwei Xia^1^, Jie Jian^1^, Yibo He^3, 4,^* Junjie Wang^2^, Pengfei Guo^1,4,^* and Hongqiang Wang^1,^*

^1^State Key Laboratory of Solidification Processing, Center for Nano Energy Materials, School of Materials Science and Engineering, Northwestern Polytechnical University and Shaanxi Joint Laboratory of Graphene (NPU), Xi’an 710072, P. R. China

^2^State Key Laboratory of Solidification Processing, School of Materials Science and Engineering Department, Northwestern Polytechnical University, Xi’an 710072, P. R. China

^3^State Key Laboratory of Solidification Processing, Center of Advanced Lubrication and Seal Materials, School of Materials Science and Engineering, Northwestern Polytechnical University, Xi’an, Shaanxi 710072, P. R. China

^4^Research& Development Institute of Northwestern Polytechnical University in Shenzhen, Shenzhen, 518063, P. R. China

^5^Key Laboratory of Applied Surface and Colloid Chemistry, Shaanxi Key Laboratory for Advanced Energy Devices, Shaanxi Engineering Lab for Advanced Energy Technology, School of Materials Science and Engineering, National Ministry of Education, Shaanxi Normal University, Xi'an 710119, P. R. China

‡ Zelin Ma and Shiyao Wang contributed equally to this work.

*Corresponding authors. E-mail: [heyibo@nwpu.edu.cn](mailto:heyibo@nwpu.edu.cn) (Yibo He); [guopengfei@nwpu.edu.cn](mailto:guopengfei@nwpu.edu.cn) (Pengfei Guo); [hongqiang.wang@nwpu.edu.cn](mailto:hongqiang.wang@nwpu.edu.cn) (Hongqiang Wang)

**Supplementary Figures and Tables**


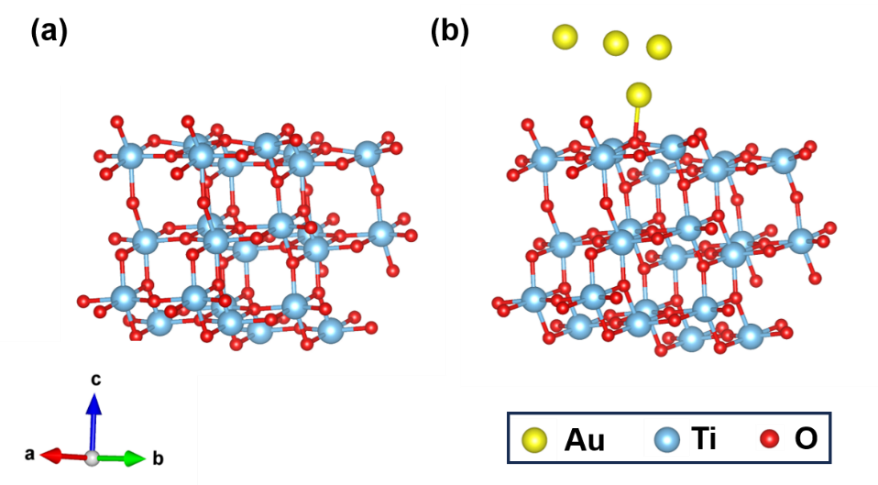


**Fig. S1** Optimized structure models of (**a**) TiO_2_ and (**b**) Au-TiO_2_


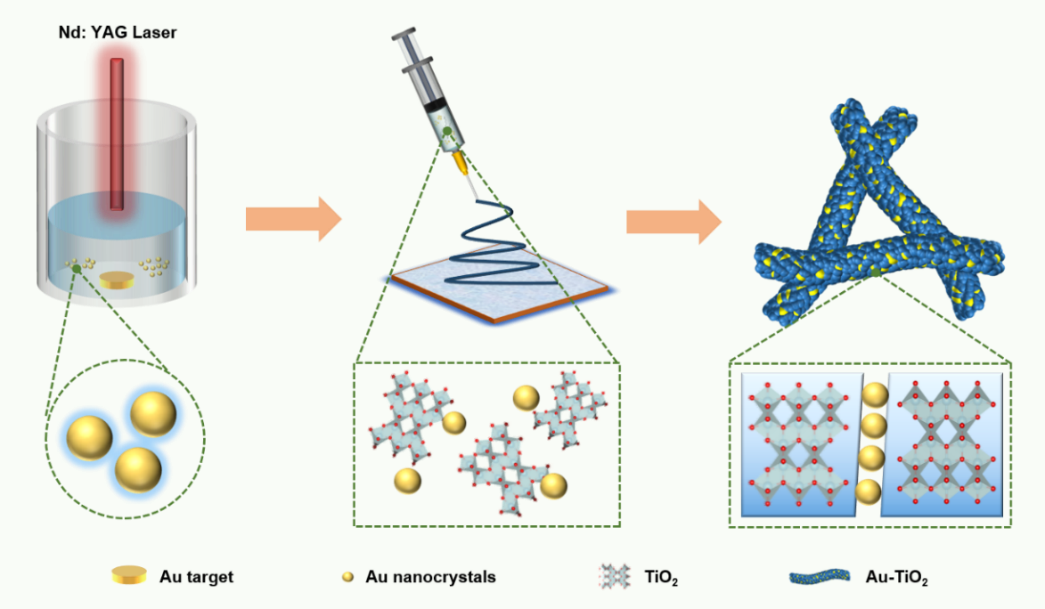


**Fig. S2** Schematic diagram of preparation process for Au nanocrystals-embedded TiO_2_ nanofibers


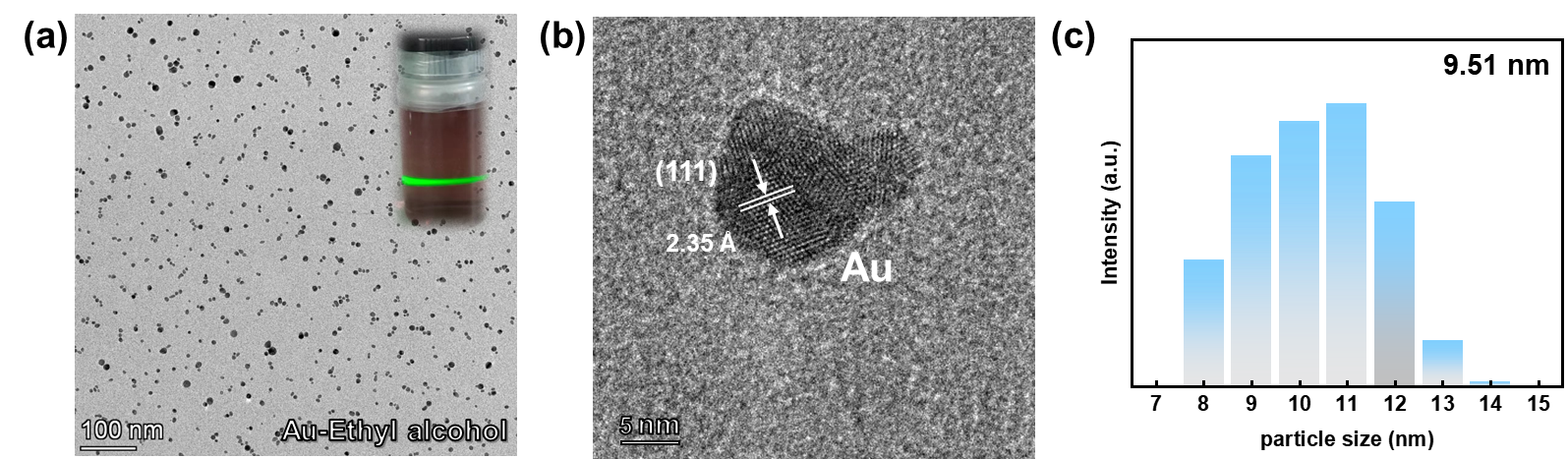


**Fig. S3** (**a**) TEM image of Au nanocrystals prepared by laser irradiation (insert, Mie-scattering image of laser-manufactured Au colloidal solution), (**b**) HRTEM image of Au nanocrystal and (**c**) particle size distribution histogram of Au nanocrystals


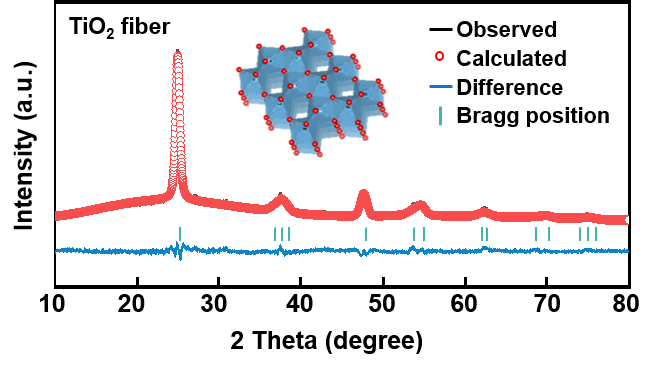


**Fig. S4** XRD patterns and Rietveld plots of TiO_2_


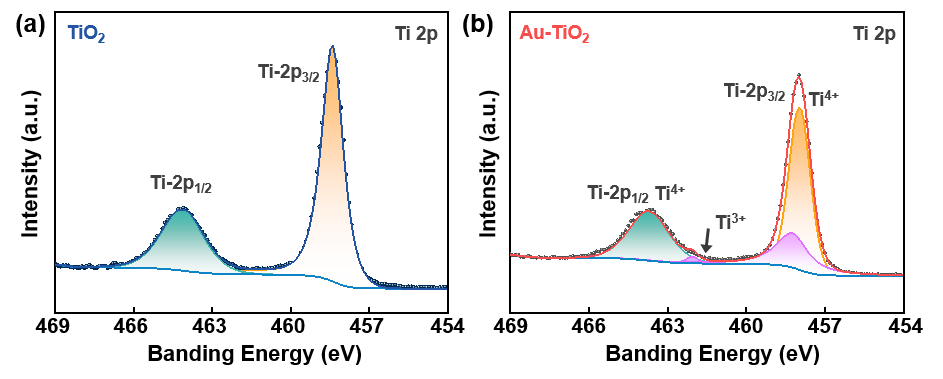


**Fig. S5** XPS spectra of Ti-2p peaks of TiO_2_ and Au-TiO_2_


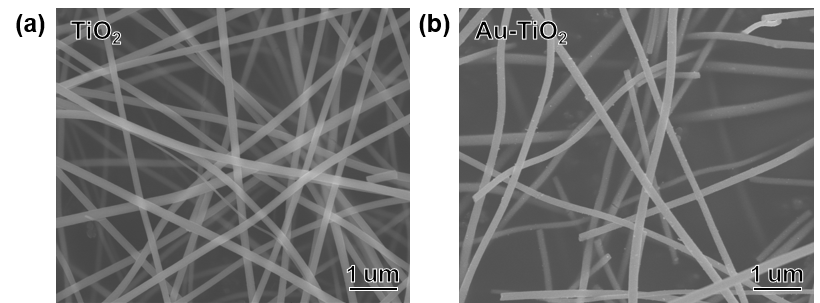


**Fig. S6** SEM images of TiO_2_ and Au-TiO_2_ nanofibers


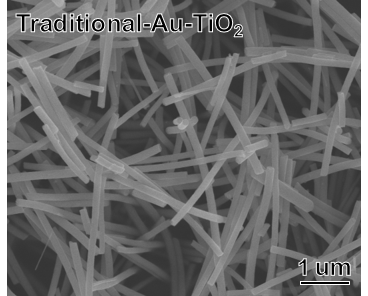


**Fig. S7** SEM image of TiO_2_ nanofibers with Au nanoparticles prepared by the traditional method with surfactants


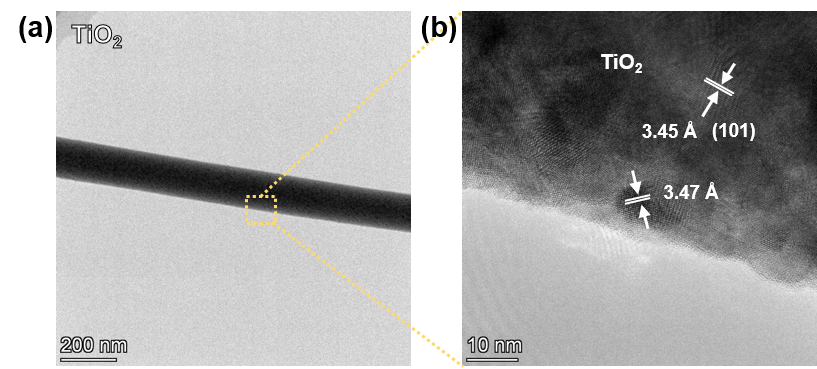


**Fig. S8** TEM and HRTEM images of TiO_2_ nanofibers


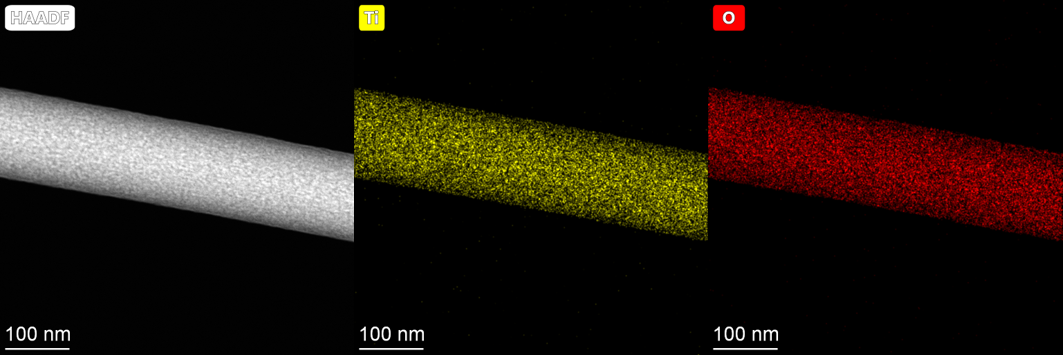


**Fig. S9** EDX mapping images of TiO_2_ nanofibers (Ti and O elements)


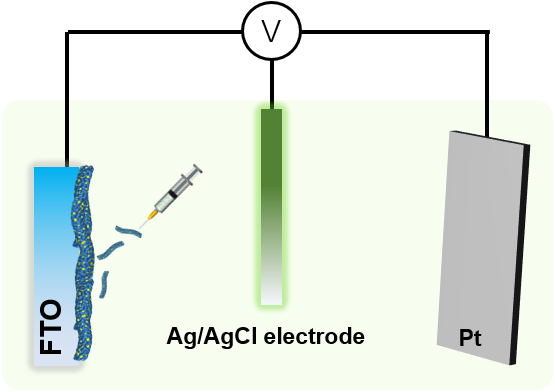


**Fig. S10** Schematic diagram of the photodetector measurement system


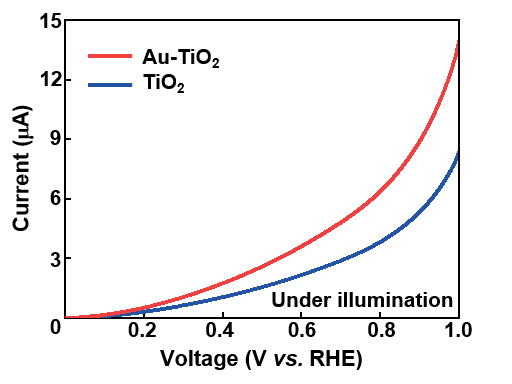


**Fig. S11** LSV curves of TiO_2_ and Au-TiO_2_ in illuminated conditions


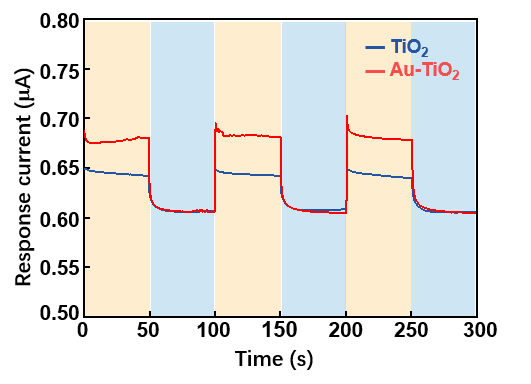


**Fig. S12** I-t curves of TiO_2_ and Au-TiO_2_ in illumination conditions at an external bias voltage of 1V


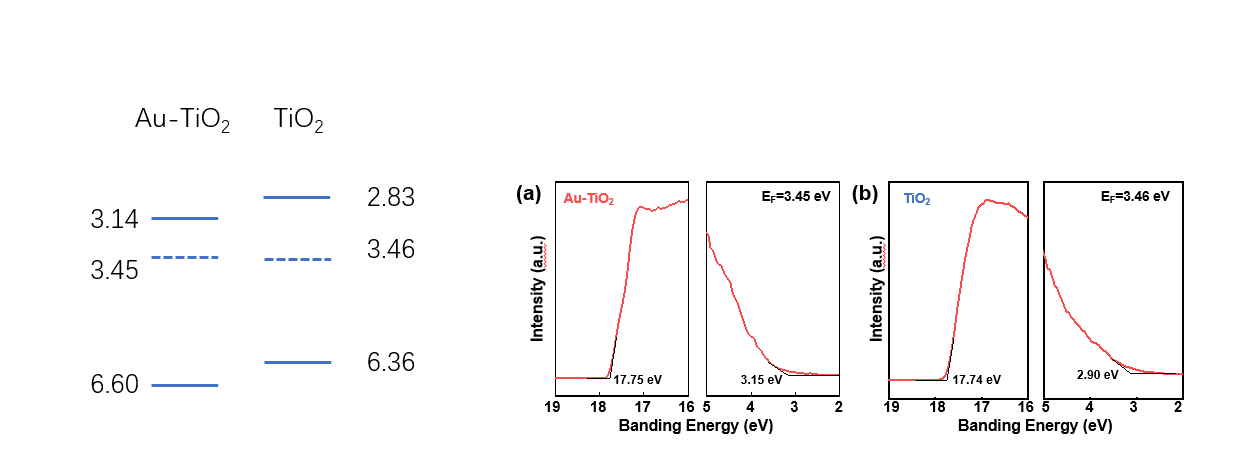


**Fig. S13** UPS spectra of (**a**) Au-TiO_2_ and (**b**) TiO_2_


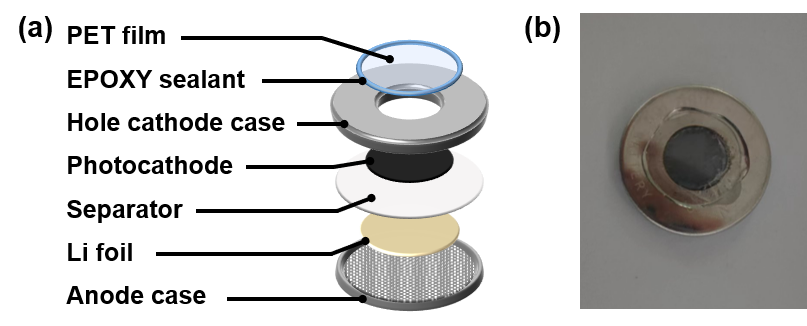


**Fig. S14** Structure diagram (**a**) and optical photograph (**b**) of photo-assisted coin cell


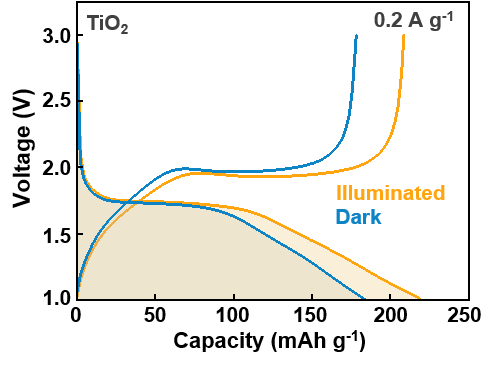


**Fig. S15** Discharge-charge curves of TiO_2_ samples at 0.2 A g^-1^ in the dark and light conditions


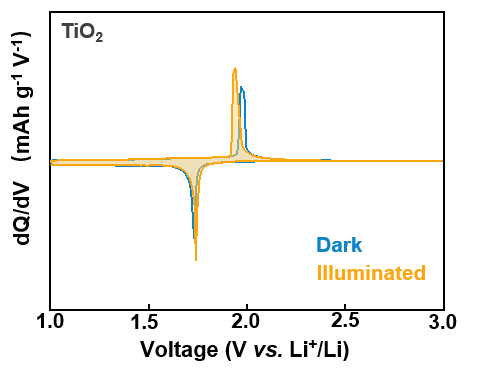


**Fig. S16** dQ/dV versus voltage of TiO_2_ samples at 0.2 A g^-1^ in the dark and light conditions


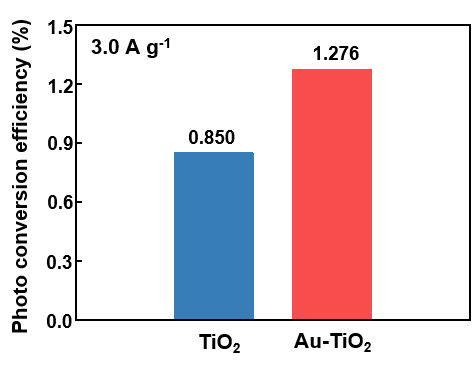


**Fig. S17** Photo-conversion efficiency of TiO_2_ and Au-TiO_2_ photocathodes under illumination at 3.0 A g^-1^


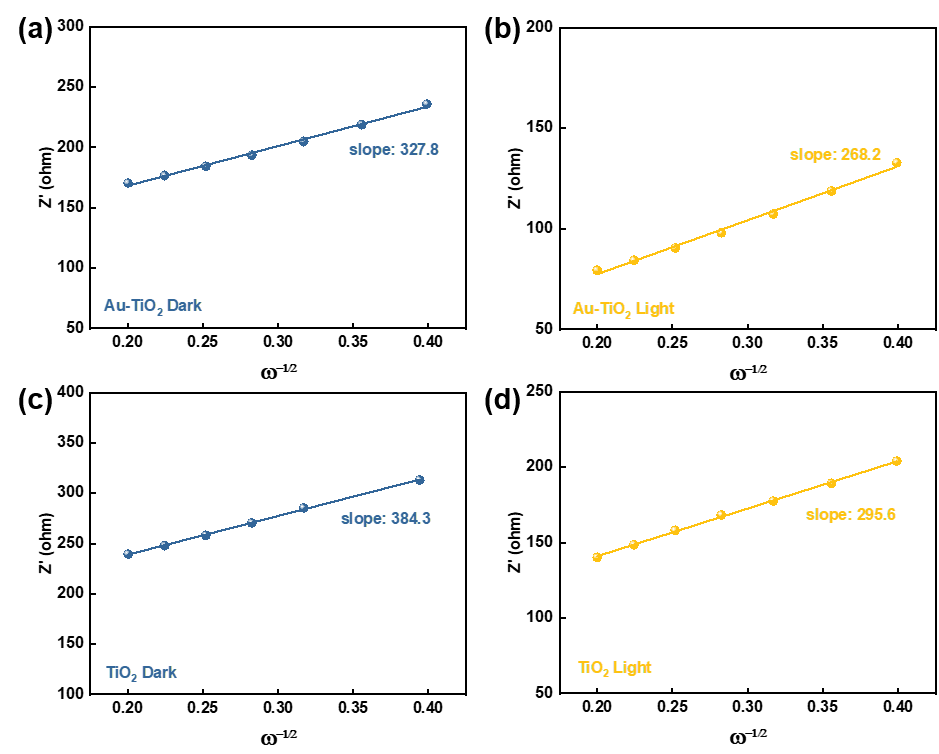


**Fig. S18** Z′ vs ω^−1/2^ curves of TiO_2_ and Au-TiO_2_ in the dark and light conditions


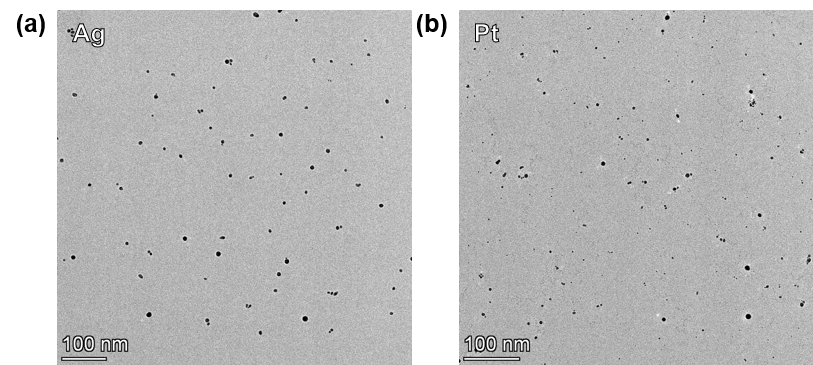


**Fig. S19** TEM images of Ag (**a**) and Pt (**b**) nanocrystals prepared by laser irradiation


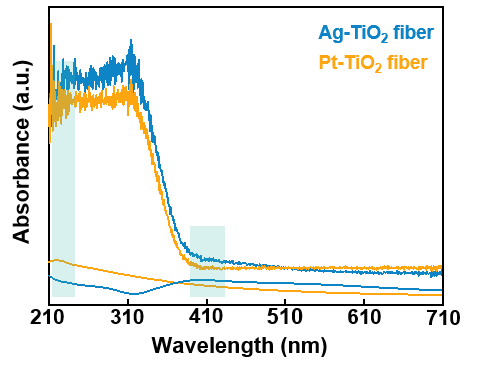


**Fig. S20** UV-vis absorption spectra of the Ag nanocrystals, Pt nanocrystals, Ag-TiO_2_ nanofiber and Pt-TiO_2_ nanofiber


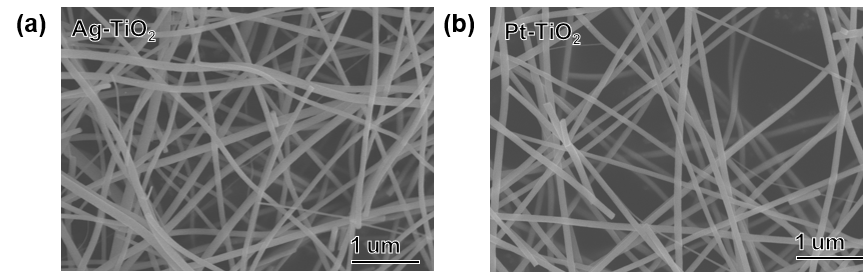


**Fig. S21** SEM images of (**a**) Ag-TiO_2_ and (**b**) Pt-TiO_2_ nanofibers


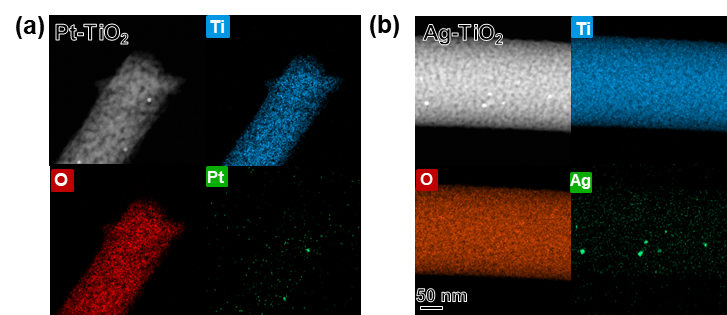


**Fig. S22** EDX mapping images of (**a**) Pt-TiO_2_ nanofiber (Ti, O and Pt elements) and (**b**) Ag-TiO_2_ nanofiber (Ti, O and Ag elements)

**Table S1** Refinement results of TiO_2_ and Au-TiO_2_

| Phase |  | TiO_2_ | Au-TiO_2_ |
| --- | --- | --- | --- |
| Symmetry |  | anatase | anatase |
| Space group |  | I41/amd | I41/amd |
| Cell parameters | a(Å) | 3.7771 (1) | 3.7779 (0) |
|  | b(Å) | 3.7771 (1) | 3.7779 (0) |
|  | c(Å) | 9.4550 (3) | 9.4559 (1) |
|  | α(°) | 90 (0) | 90 (0) |
|  | β(°) | 90 (0) | 90 (0) |
|  | γ(°) | 90 (0) | 90 (0) |
|  | V (Å^3^) | 134.892 (1) | 135.013 (0) |
|  | R*_wp_*(%) | 6.61 | 7.23 |
| Agreement factors | R*_p_*(%) | 5.18 | 5.52 |
|  | χ^2^ | 1.536 | 1.668 |

**Table S2** ICP data of Au-TiO_2_

| Sample | Au | Ti | O |
| --- | --- | --- | --- |
| Au-TiO_2_ | ~4.1% | ~95.9 % | / |
| TiO_2_ | / | ~100 % | / |

**Table S3** Specific capacities of TiO_2_ and Au-TiO_2_ in dark and light condition at different current densities

| Current density | Capacity  (mAh g^-1^) | TiO_2_ | | Au-TiO_2_ | |
| --- | --- | --- | --- | --- | --- |
|  |  | Dark | Illuminated | Dark | Illuminated |
| 0.2 A g^-1^ | discharge | 185 | 218 (18 %) | 203 | 276 (34 %) |
|  | charge | 178 | 208 | 199 | 267 |
| 0.5 A g^-1^ | discharge | 165 | 198 (21 %) | 182 | 251 (38 %) |
|  | charge | 154 | 187 | 175 | 243 |
| 1 A g^-1^ | discharge | 144 | 182 (27 %) | 164 | 233 (42 %) |
|  | charge | 136 | 175 | 158 | 226 |
| 2 A g^-1^ | discharge | 125 | 162 (30 %) | 143 | 204 (45 %) |
|  | charge | 116 | 151 | 135 | 197 |
| 3 A g^-1^ | discharge | 88 | 121 (37 %) | 105 | 163 (55 %) |
|  | charge | 79 | 112 | 96 | 153 |

**Table S4** Discharge-charge voltages of TiO_2_ and Au-TiO_2_ in dark and light condition at different current densities

| Current density | Voltage (V) | TiO_2_ | | Au-TiO_2_ | |
| --- | --- | --- | --- | --- | --- |
|  |  | Dark | Illuminated | Dark | Illuminated |
| 0.2 A g-1 | discharge | 1.710 | 1.741 | 1.738 | 1.755 |
|  | charge | 1.967 | 1.931 | 1.958 | 1.896 |
| 0.5 A g-1 | discharge | 1.710 | 1.727 | 1.715 | 1.745 |
|  | charge | 1.988 | 1.959 | 1.980 | 1.918 |
| 1 A g-1 | discharge | 1.696 | 1.721 | 1.698 | 1.739 |
|  | charge | 2.053 | 2.008 | 2.046 | 1.954 |
| 2 A g-1 | discharge | 1.644 | 1.663 | 1.655 | 1.693 |
|  | charge | 2.110 | 2.071 | 2.100 | 2.030 |

**Table S5** Photo-conversion efficiency of Au-TiO_2_ photocathodes under dark and illuminated at different current densities

| Current density (A g-1) | Charge capacity (mAh g^-1^) | | Energy (mW h) | | Illuminated  Time (h) | Photo-coversion efficiency (%) |
| --- | --- | --- | --- | --- | --- | --- |
|  | Dark | Illuminated | Dark | Illuminated |  |  |
| 0.2 | 199 | 267 | 0.239 | 0.267 | 1.34 | 0.042 |
| 0.5 | 175 | 243 | 0.210 | 0.243 | 0.49 | 0.135 |
| 1.0 | 158 | 226 | 0.190 | 0.226 | 0.23 | 0.313 |
| 2.0 | 135 | 197 | 0.162 | 0.207 | 0.11 | 0.818 |
| 3.0 | 96 | 153 | 0.115 | 0.153 | 0.06 | 1.267 |

**Table S6** Photo-conversion efficiency of TiO_2_ photocathodes under dark and illuminated at different current densities

| Current density (A g-1) | Charge capacity (mAh g^-1^) | | Energy (mW h) | | Illuminated  Time (h) | Photo-coversion efficiency (%) |
| --- | --- | --- | --- | --- | --- | --- |
|  | Dark | Illuminated | Dark | Illuminated |  |  |
| 0.2 | 178 | 208 | 0.214 | 0.228 | 1.04 | 0.027 |
| 0.5 | 154 | 187 | 0.185 | 0.196 | 0.38 | 0.058 |
| 1.0 | 136 | 175 | 0.163 | 0.175 | 0.18 | 0.133 |
| 2.0 | 116 | 151 | 0.139 | 0.151 | 0.08 | 0.300 |
| 3.0 | 79 | 112 | 0.095 | 0.112 | 0.04 | 0.850 |

**Table S7** EIS fitting data of TiO_2_ and Au-TiO_2_ in the dark and light condition

|  | Rs (Ω) | Rct (Ω) | DLi+ (cm^2^ s^-1^) |
| --- | --- | --- | --- |
| TiO2 (dark) | 3.35 | 232.1 | 4.25×10-8 |
| TiO2 (light) | 2.03 | 98.4 | 7.18×10-8 |
| Au-TiO2 (dark) | 2.22 | 136.6 | 5.84×10-8 |
| Au-TiO2 (light) | 1.49 | 45.0 | 8.72×10-8 |

**Table S8** Comparison of this work and other related work in literatures

| Number | Sample | Type | Specific capacity | Rate | Light | References |
| --- | --- | --- | --- | --- | --- | --- |
| 1 | TiO_2_ | anatase/rutile | 180 mAh g^-1^ (0.2 A g^-1^) | 180 mAh g^-1^~140 mAh g^-1^ (0.2~3 A g^-1^) | / | [S1] |
| 2 | TiO_2_ | anatase | 110 mAh g^-1^ (0.2 A g^-1^) | 110 mAh g^-1^~45 mAh g^-1^ (0.2~2 A g^-1^) | / | [S2] |
| 3 | TiO_2_ | anatase | 130 mAh g^-1^ (0.15 A g^-1^) | 130 mAh g^-1^~85 mAh g^-1^ (0.15~0.6 A g^-1^) | / | [S3] |
| 4 | TiO_2_ | anatase | 150 mAh g^-1^ (0.5 A g^-1^) | 130 mAh g^-1^~115 mAh g^-1^ (0.5~1.5 A g^-^) | / | [S4] |
| 5 | TiO_2_ | anatase | 90 mAh g^-1^ (0.15 A g^-1^) | 90 mAh g^-1^~25 mAh g^-1^ (0.15~3 A g^-1^) | / | [S5] |
| 6 | TiO_2_ | anatase | 210 mAh g^-1^ (0.2 A g^-1^) | 210 mAh g^-1^~160 mAh g^-1^ (0.2~2 A g^-1^) | / | [S6] |
| 7 | TiO_2_ | anatase | 160 mAh g^-1^ (0.1 A g^-1^) | 160 mAh g^-1^~50 mAh g^-1^ (0.1~3 A g^-1^) | / | [S7] |
| 8 | Ag/TiO_2_ | anatase | 150 mAh g^-1^ (0.1 A g^-1^) | 150 mAh g^-1^~55 mAh g^-1^ (0.1~1 A g^-1^) | / | [S8] |
| 9 | TiO_2_ | anatase | 140 mAh g^-1^ (0.15 A g^-1^) | 140 mAh g^-1^~55 mAh g^-1^ (0.15~1.5 A g^-1^) | / | [S9] |
| 10 | TiO_2_ | anatase | 130 mAh g^-1^ (1.84 uA cm^-2^) | / | / | [S10] |
| 11 | TiO_2_ | anatase | 190 mAh g^-1^ （0.3 A g^-1^） | / | 230 mAh g^-1^ (0.3 A g^-1^) | [S11] |
| 12 | TiO_2_ | anatase | 185 mAh g^-1^ (0.2 A g^-1^) | 185mAh g^-1^~88 mAh g^-1^ (0.2 A g^-1^~3 A g^-1^) | 218 mAh g^-1^~121 mAh g^-1^ (0.2 A g^-1^~3 A g^-1^) | This work |
| 13 | Au-TiO_2_ | anatase | 205 mAh g^-1^ (0.2 A g^-1^) | 205 mAh g^-1^~105 mAh g^-1^ (0.2 A g^-1^~3 A g^-1^) | 276 mAh g^-1^~163 mAh g^-1^ (0.2 A g^-1^~3 A g^-1^) | This work |

**Supplementary References**

1. W. Song, Q. Jiang, X. Xie, A. Brookfield, E.J.L. McInnes et al., Synergistic storage of lithium ions in defective anatase/rutile TiO_2_ for high-rate batteries. Energy Storage Mater. **22**, 441-449 (2019). <https://doi.org/10.1016/j.ensm.2019.07.025>
2. Y. Chen, J. Chen, J. Liu, Z. Lin, X. Hu et al., Metal-organic framework-derived mixed-phase anatase/rutile TiO_2_ towards boosted lithium storage: Surface engineering and design strategy through crystal phase transition. Mater. Today Nano **22**, 100265 (2022). <https://doi.org/10.1016/j.mtnano.2022.100265>
3. J. Ma, K.G. Reeves, A.-G.P. Gutierrez, M. Body, C. Legein et al., Layered lepidocrocite type structure isolated by revisiting the sol–gel chemistry of anatase TiO_2_: A new anode material for batteries. Chem. Mater. **29**, 8313-8324 (2017). <https://doi.org/10.1021/acs.chemmater.7b02674>
4. Y. Cai, H.-E. Wang, X. Zhao, F. Huang, C. Wang et al., Walnut-like porous core/shell TiO_2_ with hybridized phases enabling fast and stable lithium storage. ACS Appl. Mater. Interfaces **9**, 10652 (2017). <https://doi.org/10.1021/acsami.6b16498>
5. M. Zhang, K. Yin, Z.D. Hood, Z. Bi, C.A. Bridges et al., In situ TEM observation of the electrochemical lithiation of N-doped anatase TiO_2_ nanotubes as anodes for lithium-ion batteries. J. Mater. Chem. A **5**, 20651-20657 (2017). <https://doi.org/10.1039/C7TA05877B>
6. D.-H. Lee, B.-H. Lee, A.K. Sinha, J.-H. Park, M.-S. Kim et al., Engineering titanium dioxide nanostructures for enhanced lithium-ion storage. J. Am. Chem. Soc. **140**, 16676-16684 (2018). <https://doi.org/10.1021/jacs.8b09487>
7. Y. Qiu, K. Yan, S. Yang, L. Jin, H. Deng et al., Synthesis of size-tunable anatase TiO_2_ nanospindles and their assembly into anatase@titanium oxynitride/titanium nitride−graphene nanocomposites for rechargeable lithium ion batteries with high cycling performance. ACS Nano **11**, 6515-6526 (2010). <https://doi.org/10.1021/nn101603g>
8. Y. Zhang, J. Li, W. Li, D. Kang, Synthesis of one-dimensional mesoporous Ag nanoparticles-modified TiO_2_ nanofibers by electrospinning for lithium ion batteries. Materials **12**, 2630 (2019). <https://doi.org/10.3390/ma12162630>
9. D.T. D, S. Mohapatra, S.V. Nair, A.S. Nair, A.K. Ra, Surfactant-assisted synthesis of porous TiO_2_ nanofibers as an anode material for secondary lithium ion batteries. Sustainable Energy Fuels **1**, 138-144 (2017). <https://doi.org/10.1039/C6SE00030D>
10. D. Imazeki, C.C. Gils, K. Nishio, R. Shimizu, T. Hitosugi, Effects of anisotropy in rutile TiO_2_ on the performance of solid-state lithium batteries. ACS Appl. Energy Mater. **3**, 8338-8343 (2020). <https://doi.org/10.1021/acsaem.0c00910>
11. O. Nguyen, E. Courtin, F. Sauvage, N. Krins, C. Sancheza et al., Shedding light on the light-driven lithium ion de-insertion reaction: towards the design of a photo-rechargeable battery. J. Mater. Chem. A **5**, 5927-5933 (2017). <https://doi.org/10.1039/C7TA00493A>
